# Comparative analysis of Cochrane and non-Cochrane reviews over three decades

**DOI:** 10.1186/s13643-024-02531-2

**Published:** 2024-05-02

**Authors:** Mikkel Zola Andersen, Philine Zeinert, Jacob Rosenberg, Siv Fonnes

**Affiliations:** 1 Center for Perioperative Optimization, Department of Surgery, Herlev and Gentofte Hospitals, University of Copenhagen, Borgmester Ib Juuls Vej 1, Herlev, 2730 Denmark; 2 Cochrane Colorectal Group, Herlev and Gentofte Hospitals, University of Copenhagen, Borgmester Ib Juuls Vej 1, Herlev, 2730 Denmark; 3 Copenhagen University Library, Royal Danish Library, Søren Kierkegaards Plads 1, Copenhagen K, 1221 Denmark

**Keywords:** Systematic review, Cochrane, Evidence-based medicine, Information science, Bibliometrics

## Abstract

**Background:**

Systematic reviews are viewed as the best study design to guide clinical decision-making as they are the least biased publications assuming they are well-conducted and include well-designed studies. Cochrane was initiated in 1993 with an aim of conducting high-quality systematic reviews. We aimed to examine the publication rates of non-Cochrane systematic reviews (henceforth referred to simply as “systematic reviews”) and Cochrane reviews produced throughout Cochrane’s existence and characterize changes throughout the period.

**Methods:**

This observational study collected data on systematic reviews published between 1993 and 2022 in PubMed. Identified Cochrane reviews were linked to data from the Cochrane Database of Systematic Reviews via their Digital Object Identifier. Systematic reviews and Cochrane reviews were analyzed separately. Two authors screened a random sample of records to validate the overall sample, providing a precision of 98%.

**Results:**

We identified 231,602 (94%) systematic reviews and 15,038 (6%) Cochrane reviews. Publication of systematic reviews has continuously increased with a median yearly increase rate of 26%, while publication of Cochrane reviews has decreased since 2015. From 1993 to 2002, Cochrane reviews constituted 35% of all systematic reviews in PubMed compared with 3.5% in 2013–2022. Systematic reviews consistently had fewer authors than Cochrane reviews, but the number of authors increased over time for both. Chinese first authors conducted 15% and 4% of systematic reviews published from 2013–2022 and 2003–2012, respectively. Most Cochrane reviews had first authors from the UK (36%). The native English-speaking countries the USA, the UK, Canada, and Australia produced a large share of systematic reviews (42%) and Cochrane reviews (62%). The largest publishers of systematic reviews in the last 10 years were gold open access journals.

**Conclusions:**

Publication of systematic reviews is increasing rapidly, while fewer Cochrane reviews have been published through the last decade. Native English-speaking countries produced a large proportion of both types of systematic reviews. Gold open access journals and Chinese first authors dominated the publication of systematic reviews for the past 10 years. More research is warranted examining why fewer Cochrane reviews are being published. Additionally, examining these systematic reviews for research waste metrics may provide a clearer picture of their utility.

**Supplementary Information:**

The online version contains supplementary material available at 10.1186/s13643-024-02531-2.

## Introduction

The amount of scientific literature is growing exponentially [1]. With the introduction of evidence-based medicine in the late part of the twentieth century, synthesizing evidence became an area of focus [2]. The principles of evidence-based medicine dictate that the best clinical guidance should be based on the least biased and most up-to-date evidence available. This comes in the form of systematic reviews and meta-analyses, which are generally viewed as the study design that is least likely to be biased, assuming they are well-conducted and based on methodologically rigorous studies [3], and thereby providing the best evidence for informing decision-makers [4, 5]. In 1993, the Cochrane Collaboration (now “Cochrane”) was founded on the same ideals as evidence-based medicine was built upon, with an aim of providing systematic reviews and meta-analyses of high quality [6]. While there are examples of synthesizing evidence systematically several hundred years ago [7, 8], the development of the methodology and frequency of publication of systematic reviews has accelerated during the last 30 years where Cochrane has existed. Cochrane has played a considerable role in developing a thorough systematic review methodology [9, 10] and creating software to ease the production of systematic reviews, e.g., RevMan Web (Review Manager Web, version 4.12.0, The Cochrane Collaboration, 2022). Other non-Cochrane initiatives to streamline and improve systematic review methodology have also been developed, e.g., the Meta-analyses of Observational Studies in Epidemiology (MOOSE) guideline [11], the Preferred Reporting Items for Systematic reviews and Meta-Analyses (PRISMA) guideline [12], and the Joanna Briggs Institute guidelines [13]. However, with the development and distribution of systematic review methodology, it is unclear how the distribution of systematic review production through the years has changed.

We aimed to characterize changes in publication of non-Cochrane systematic reviews (henceforth referred to simply as “systematic reviews”) and Cochrane reviews throughout Cochrane’s existence. Our primary objective was to determine the yearly publication rates of systematic reviews and Cochrane reviews through a 30-year period from 1993 to 2022. Our secondary outcome was to further characterize changes for both types of reviews.

## Methods

### Reporting, eligibility criteria, and validation

This observational study was reported according to the RECORD guideline where applicable [14] as it was based on routinely collected metadata (Appendix). Records were eligible for inclusion if they were any type of systematic reviews published between 1993 and 2022 and indexed in PubMed or the Cochrane Database of Systematic Reviews (CDSR). All types of published systematic reviews were included, i.e., also updated Cochrane reviews, withdrawn Cochrane reviews [15], and retracted systematic reviews. We chose this period as it was the time from the creation of the CDSR until the last full calendar year before our date of search. We excluded records with missing data such as title, journal, or year; records with publication date registered before 1993 or in 2023; protocols of Cochrane reviews, which was assessed via metadata from the CDSR regarding the stage of the report; duplicate records; and protocols of systematic reviews by searching the title of records for the terms “review protocol,” “protocol for a systematic,” “protocol of a systematic,” “protocol for the systematic,” “protocol of the systematic,” “protocol for systematic,” and “protocol of systematic.” The terms for systematic review protocols were determined by pilot searches. We performed additional pilot investigations of the extracted data to determine the best options for data cleaning. As our pilot investigations indicated errors in the indexation of publication date for early Cochrane reviews, we matched Cochrane reviews from PubMed to the CDSR based on their Digital Object Identifier (DOI) and collected data on publication date from the CDSR. The country of the first author was extracted in an automated fashion from the registered first author affiliation from the PubMed metadata. All countries that appeared over 100 times after data cleaning were validated manually and integrated into the analysis. Furthermore, for the Cochrane reviews, we screened all records with more than one country mentioned in the affiliations and looked up unresolved countries to integrate these into the final analysis. For further validation, we screened a random sample of 1182 without replacement of the extracted records (0.5%) and determined a precision [16]. Two authors independently screened the 1182 random records from the PubMed sample for relevance. Relevant records were defined as either identifying as a systematic review or conducting a literature review via a systematic search. Records were initially screened on title, subsequently abstract, and finally full text if needed. Conflicts were resolved through discussion between the two authors. In total, 21 records from the sample were irrelevant correlating to a precision of 98%. We found no sign of systematic errors among the 21 ineligible records, thus, there was no further exclusion of records.

### Data sources, linkage, variables, and study size

Data were obtained through an Application Programming Interface (API) and Entrez Direct Server, for PubMed [17], and linked with the CDSR [18] via the Cochrane Reviews’ unique DOI. PubMed is the most used biomedical scholarly database and provides good coverage of biomedical literature [19]. At the time of data extraction, the systematic review filter in PubMed was accurate and up to date (S Schmidt, Senior Technical Information Specialist, National Library of Medicine, personal communication, 04 April 2023). The search of PubMed was done via Entrez Direct Server on 26 April 2023. We extracted data on systematic review characteristics (title, authors, publication date, journal, International Standard Serial Number (ISSN), affiliations, funding, conflicts of interest statement, and DOI) through indexed metadata in PubMed via the API and from the CDSR. During reporting, countries were abbreviated following the United Nations Conference on the Standardization of Geographical Names abbreviations [20]. The study size was determined by the records obtained that met our inclusion criteria and none of the exclusion criteria. We did not attempt to limit the sample.

### Statistical analyses

Data were descriptively analyzed for the full 30-year period and in three separate subgroups each covering a 10-year period:1993–2002, 2003–2012, and 2013–2022. The number of authors was handled as continuous data and was reported with mean and standard deviation. Yearly percentual increase was calculated and displayed tabulated. Systematic reviews’ and Cochrane reviews’ growth rates were presented graphically by scaling the increases of the yearly number of reviews to a shared range between 0 and 1 by unity-based normalization. Other data were handled as categorical variables and reported as number and percent. Statistical analyses were done in Python v3.10, using Pyspark v3.4.1 for big data handling and several standard libraries like NumPy v1.22.4, Pandas v1.5.3, Scikit-Learn v1.2.2, SciPy v1.10.1, and matplotlib v3.7.1 for calculations and visualizations. Codes are available upon reasonable request.

## Results

We extracted 264,127 records from PubMed. Two records were excluded as they had no registered date. Subsequently, 15,330 records were excluded for being published either before 1993 (*n* = 33) or in 2023 (*n* = 15,297), and 1344 were excluded for being protocols of systematic reviews. After a manual assessment of DOI duplicates, further 50 records were excluded. After DOI-matching to the CDSR, a total of 246,640 records were analyzed, of which 231,602 (93.9%) were systematic reviews and 15,038 (6.1%) were Cochrane reviews (Table 1).

**Table 1:** Characteristics of non-Cochrane systematic reviews and Cochrane reviews

Non-Cochrane systematic reviews					Cochrane reviews			
Period	1993–2002	2003–2012	2013–2022	Total	1993–2002	2003–2012	2013–2022	Total
Publications	2522 (1)	29,135 (13)	199,945 (86)	231,602	1377 (9)	6421 (43)	7240 (48)	15,038
Authors	3.6 ± 2.1	4.5 ± 2.9	5.9 ± 3.9	5.7 ± 3.8	3.2 ± 1.7	4.0 ± 1.8	5.3 ± 2.7	4.5 ± 2.4
Country	*n* = 2118	*n* = 26,690	*n* = 178,445	*n* = 207,253	*n* = 1350	*n* = 6059	*n* = 7205	*n* = 14,614
#1	GB 716 (34)	US 6172 (23)	CN 27230 (15)	US 33012 (16)	GB 574 (43)	GB 2177 (36)	GB 2462 (34)	GB 5213 (36)
#2	US 482 (23)	GB 5416 (20)	US 26358 (15)	CN 28313 (14)	AU 143 (11)	AU 777 (13)	AU 768 (11)	AU 1688 (12)
#3	CA 197 (9)	CA 2518 (9)	GB 21056 (12)	GB 27188 (13)	CA 140 (10)	CA 456 (8)	US 497 (7)	US 1072 (7)
#4	NL 182 (9)	NL 1873 (7)	AU 12103 (7)	AU 14043 (7)	US 120 (9)	US 455 (8)	CA 430 (6)	CA 1026 (7)
#5	AU 128 (6)	AU 1812 (7)	CA 9877 (6)	CA 12592 (6)	NZ 50 (4)	NL 308 (5)	CN 374 (5)	NL 657 (4)

Characteristics expressed as number (%) or mean ± standard deviation of included non-Cochrane systematic reviews and Cochrane reviews for periods of 10 years and the total 30-year period. Regarding countries, the top five most common countries of first author affiliations for the given periods were listed with the total number of systematic reviews published. Countries are abbreviated following the United Nations Conference on the Standardization of Geographical Names abbreviations [20]. Data were collected from PubMed and data on Cochrane reviews were linked via the records Digital Object Identifier. For each outcome, *n* represents how many records had data available for the outcome.

**Fig. 1:**
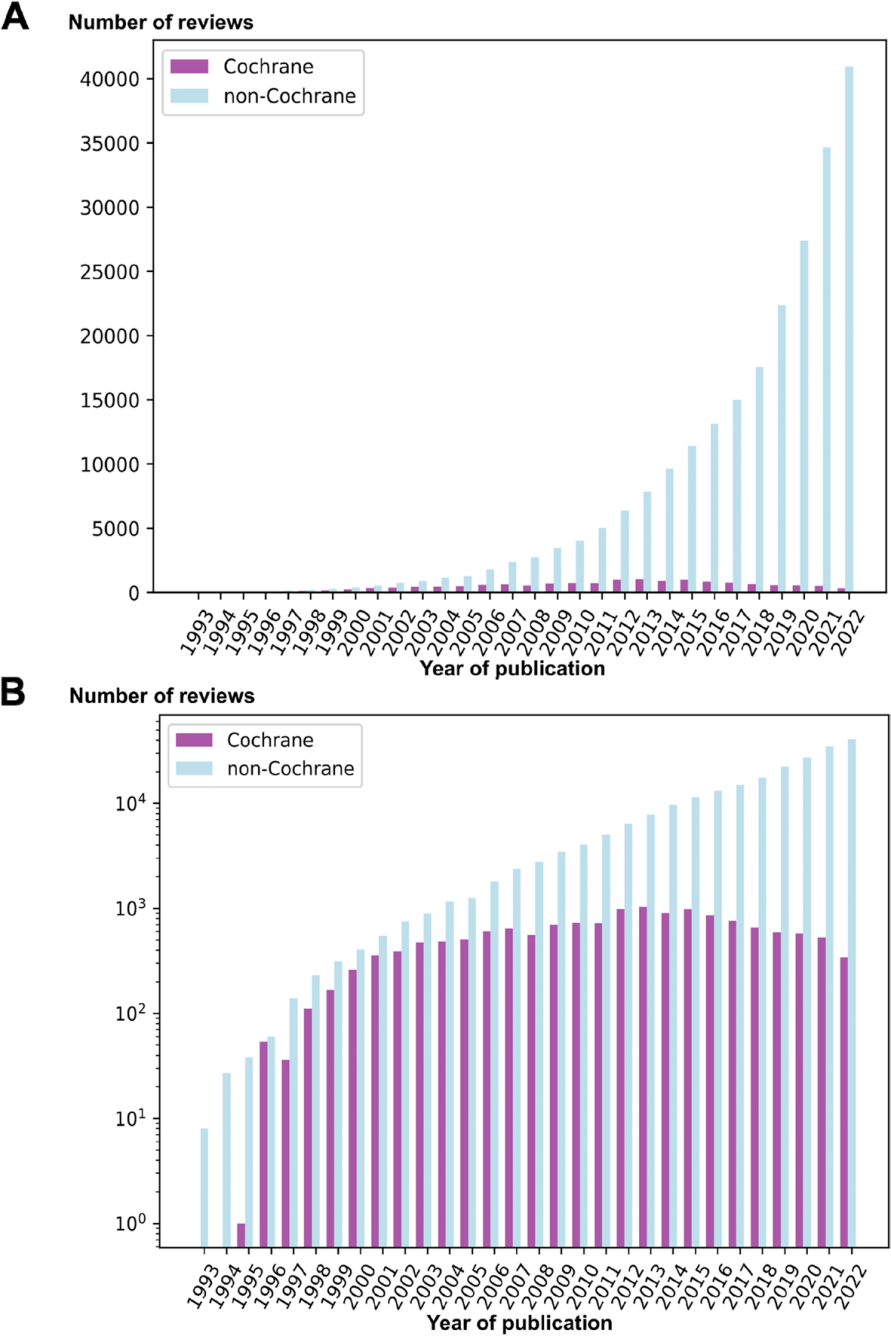
Absolute change in non-Cochrane systematic reviews and Cochrane reviews. Total number (*y*-axis) of non-Cochrane systematic reviews (light blue) indexed in PubMed and Cochrane reviews (purple) indexed in the Cochrane Database of Systematic Reviews published per year (*x*-axis) from 1993 to 2022 as absolute number (**A**) and expressed logarithmically (**B**)

**Fig. 2:**
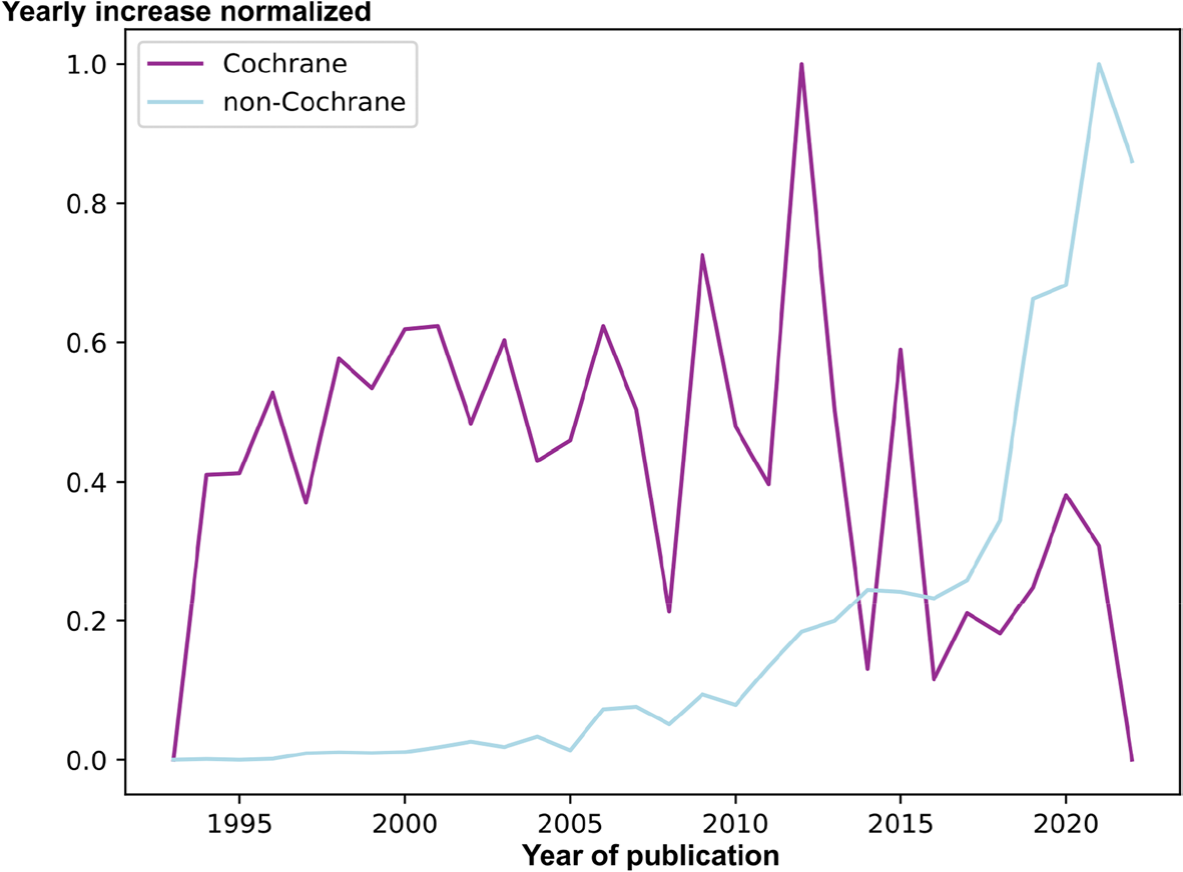
Normalized yearly change of non-Cochrane systematic reviews and Cochrane reviews. Unity-based normalization in a range of [0, 1] of absolute yearly changes in non-Cochrane systematic reviews and Cochrane reviews

The number of published systematic reviews increased continuously throughout the 30-year period (Fig. 1) with a median yearly increase rate of 26% (Table 2). Meanwhile, Cochrane reviews reached their peak in productivity in 2013 and growth rates fluctuated over the whole 30-year period with a median yearly increase of 3%. Since 2015, Cochrane reviews have been published less frequently (Figs. 1, 2 and Table 2). For the period 1993–2002, 1377 Cochrane reviews were published, constituting 35% of all systematic reviews indexed in PubMed (Fig. 1). For the period 2013–2022, 7240 Cochrane reviews were published corresponding to 3.5% of all systematic reviews in PubMed for the period, and in 2022 alone, 344 Cochrane reviews were published, corresponding to a share of 0.8%.

**Table 2:** Yearly percentual increase or decrease (indicated by negative numbers) in publication of non-Cochrane systematic reviews and Cochrane reviews throughout the 30-year period

**Year**	**Non-Cochrane systematic reviews**		**Cochrane reviews**	
	***n***	**%**	***n***	**%**
1993	8	0	0	0
1994	27	238	0	0
1995	38	41	1	0
1996	60	58	54	5300
1997	140	133	36	− 33
1998	230	64	111	208
1999	313	36	167	50
2000	406	30	261	56
2001	549	35	357	37
2002	751	37	390	9
2003	897	19	477	22
2004	1152	28	486	2
2005	1264	10	508	5
2006	1805	43	604	19
2007	2372	31	646	7
2008	2755	16	557	− 14
2009	3451	25	699	25
2010	4037	17	730	4
2011	5024	24	724	− 1
2012	6378	27	990	37
2013	7844	23	1032	4
2014	9640	23	906	− 12
2015	11,416	18	987	9
2016	13,123	15	854	− 13
2017	15,018	14	764	− 11
2018	17,544	17	661	− 13
2019	22,383	28	588	− 11
2020	27,366	22	575	− 2
2021	34,665	27	529	− 8
2022	40,946	18	344	− 35

**Fig. 3:**
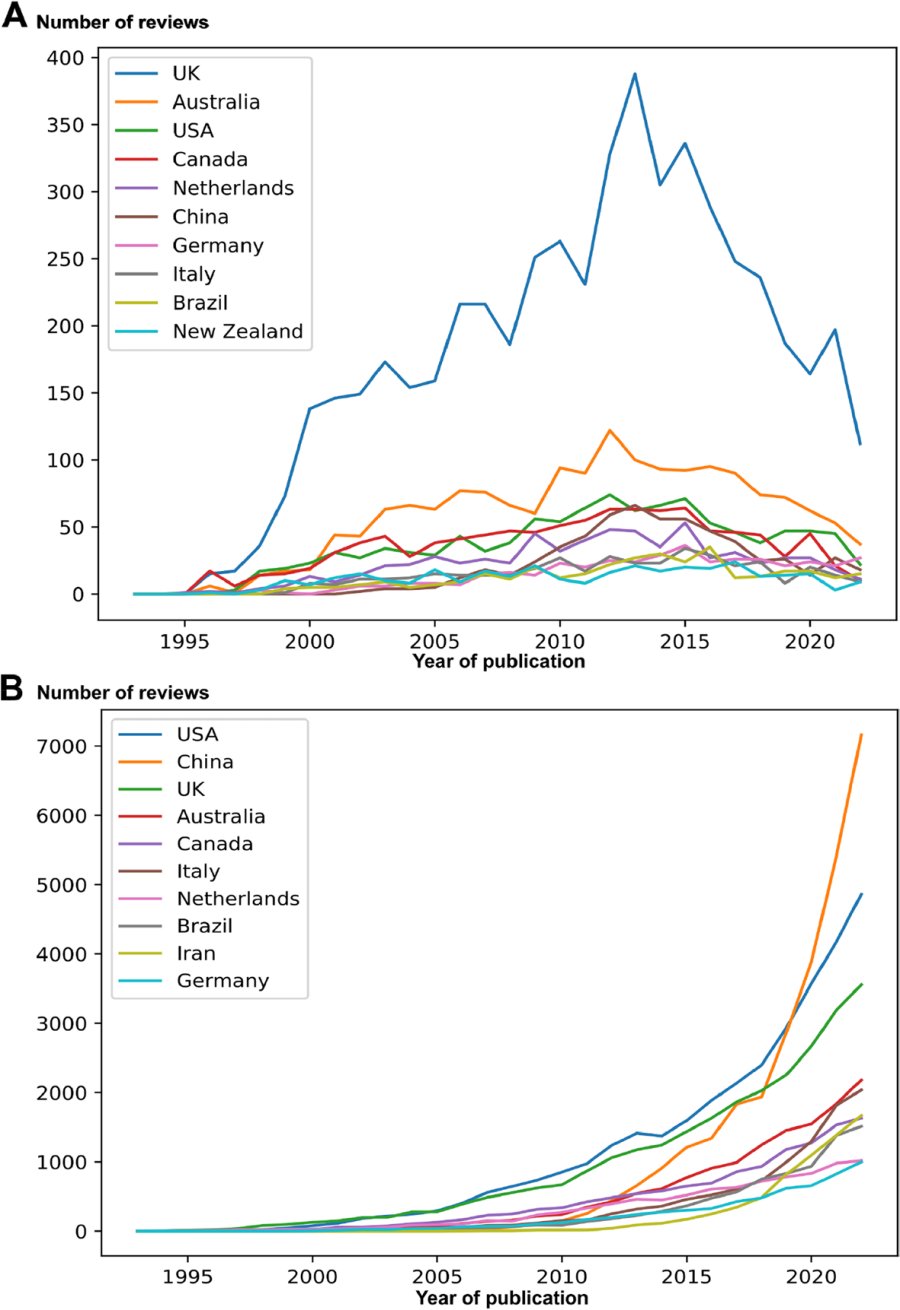
Country of first authors for non-Cochrane systematic reviews and Cochrane reviews. The plot of the number of publication (*y*-axis) of Cochrane reviews (**A**) and non-Cochrane systematic reviews (**B**) by the top 10 first-author affiliated countries from 1993 to 2022 (*x*-axis). Analysis was based on 14,614 records (97%) (**A**) and analysis was based on 207,253 records (89%) (**B**)

Systematic reviews had a mean of 3.6 authors per review in 1993–2002, which increased to 5.9 in 2013–2022. Meanwhile, Cochrane reviews had 3.2 authors in 1993–2002, which increased to 5.3 in 2013–2022. Cochrane reviews continuously had fewer authors than systematic reviews for all 10-year periods. From 2013–2022, Chinese first authors published most systematic reviews (27,230, 15%). Meanwhile, for Cochrane reviews in the same period, Chinese first authors published 5% of these. From 1993–2002 and 2003–2012, Chinese first authors published 4 (0.2%) and 1079 (4%) systematic reviews, respectively. Generally, the native English-speaking countries the USA, the UK, Canada, and Australia combined produced a large proportion of the published systematic reviews for both systematic reviews (86,835, 42%) and Cochrane reviews (8999, 62%) (Fig. 3). The UK had produced most Cochrane reviews throughout the 30-year period (5213, 36%) and for all 10-year intervals, but the share of Cochrane reviews produced by UK first authors was decreasing (Fig. 3). In the first 10 years of the period, high-impact journals such as the BMJ, JAMA, and Lancet were among the journals publishing most systematic reviews (Table 3, Additional File 1). From 2013 to 2022, gold open access journals constituted the top eight journals publishing systematic reviews, while the remaining two journals in the top ten used a hybrid open access model. In total, 8851 unique journals published systematic reviews for the 30-year period.

**Table 3:** Journals publishing non-Cochrane systematic reviews. National Library of Medicine (NLM) abbreviations of journals with most published non-Cochrane systematic reviews based on International Standard Serial Number (ISSN) expressed as follows: total number (%). For ISSN see Supplementary File 1

Rank	1993–2002 (*n* = 2522)		2003–2012 (*n* = 29,135)		2013–2022 (*n* = 199,945)		Total (*n* = 231,602)	
#1	BMJ (print)	95 (3.8)	JBI Libr Syst Rev	535 (1.8)	PloS One	4404 (2.2)	PLoS One	4680 (2.0)
#2	Health Technol Assess	91 (3.6)	BMJ Clin Evid	452 (1.6)	Medicine (Baltimore)	2263 (1.1)	Int J Environ Res Public Health	2278 (1.0)
#3	JAMA	31 (1.2)	BMJ (print)	298 (1.0)	Int J Environ Res Public Health	2262 (1.1)	Medicine (Baltimore)	2270 (1.0)
#4	Br J Gen Pract	31 (1.2)	PLoS One	276 (0.9)	BMJ Open	1735 (0.9)	BMJ Open	1765 (0.8)
#5	Lancet	30 (1.2)	Ann Intern Med	170 (0.6)	Nutrients	1234 (0.6)	Nutrients	1237 (0.5)
#6	BMJ (electronic)	27 (1.1)	Spine	157 (0.5)	J Clin Med	1040 (0.5)	J Clin Med	1040 (0.4)
#7	Spine	25 (1.0)	Health Technol Assess	154 (0.5)	Front Oncol	978 (0.5)	Front Oncol	978 (0.4)
#8	Thorax	22 (0.9)	Obes Rev	136 (0.5)	Sci Rep	927 (0.5)	Sci Rep	927 (0.4)
#9	Br J Surg	21 (0.8)	BMC Public Health	134 (0.5)	World Neurosurg	799 (0.4)	BMC Public Health	829 (0.4)
#10	Arch Intern Med	21 (0.8)	Aliment Pharmacol Ther	123 (0.4)	Cureus	775 (0.4)	World Neurosurg	802 (0.3)

## Discussion

In this observational study, we found that the number of published systematic reviews has increased considerably, whereas Cochrane’s relative share of systematic reviews has decreased throughout its existence. While systematic reviews increased, the publication of Cochrane reviews has decreased steadily since 2015. During the last decade, Chinese first authors produced a large portion of systematic reviews. Gold open access journals were the largest publishers of systematic reviews from 2013 to 2022, whereas high-impact journals were the largest publishers of systematic reviews from 1993 to 2002. The number of authors seemed to increase over time.

We found that publication of systematic reviews is growing rapidly. Compared with other general publications in life sciences, where authors found a growth rate of 5.1% [1], systematic reviews are growing faster. Our findings show that while the motivation for publishing systematic reviews was high, Cochrane reviews were being published less frequently. Cochrane reviews are known to take a long time to complete [21–23] compared with systematic reviews [24–26]. This may be partly due to the process of a Cochrane review having more mandatory steps involved, e.g., minimum requirements for databases searched, searching trial registries, and requirements regarding which analyses to conduct. This may also explain why they are known overall to be of higher quality [27–32] and are valuable for developing healthcare policies [33]. The thought of high demands for resources and long publication times may intimidate potential authors. However, despite this, Cochrane’s author satisfaction was generally high [34]. Recently, Cochrane has begun rethinking their publication model [35] with several key projects aimed at improving the author experience and optimizing the publication process [36].

We found that balances had shifted relating to production and publication of systematic reviews over the past 10 years. Regarding production, especially systematic reviews written by Chinese first authors have expanded, increasing their publication of systematic reviews by a factor of 25 from 2003–2012 to 2013–2022. The same productivity increase among Chinese authors was not seen in Cochrane reviews. Despite the rapid increase in productivity, one Chinese study found similar methodological and reporting quality between Chinese systematic reviews and those from the USA [37]. Regarding publication, gold open access journals had grown to be the largest publishers of systematic reviews from 2013 to 2022. Open access has been gaining popularity and may be a step in the right direction for science [38]. However, gold open access journals operate with article processing charges that authors must pay to get their papers published. These charges can be steep and have been increasing rapidly over time [39]. There is a wish within the scientific community to transition to a diamond open-access model, where articles are published open access but without authors or readers having to pay exorbitant fees [40]. While this is undoubtedly desirable, there are still costs associated with academic publishing and determining how the diamond open-access model should be financed is not an easy matter. With the increasing amount of research production, there has been discussion of “research waste” [41], i.e., unnecessary and non-contributing research. It was argued already in 1994, at the beginning of our inclusion period, that the scientific community should aim towards producing fewer reviews and instead focus on increasing the quality of publications [42]. Since then, we have had 30 years of exponential increase in evidence synthesis. Relating to this concept, several suggestions towards reducing redundancy of reviews have been put forward [43]. Addressing research waste seems to be a topic gaining focus [43, 44]. We also found an increasing number of authors through time. This aligns with the findings of other studies [45, 46]. This may relate to an increasing amount of data in modern research thereby increasing complexity [47]. One study, however, found that adjusting for factors relating to increased complexity did not explain the increase in authorship when it came to trials and non-randomized studies [48]. Furthermore, systematic reviews differ from clinical trials in that data are available remotely through searches, and therefore, the resource demands may be lower. Interestingly, Cochrane reviews had fewer authors than systematic reviews. This may seem counterintuitive, as Cochrane reviews generally have higher demands and should therefore demand more resources. It is not clear what is causing the increasing number of authors in medicine.

Our study had several strengths. Firstly, we analyzed a very large dataset with the goal of having generalizable conclusions. We only searched PubMed, which is a biomedical database, where records are manually screened for indexation as systematic reviews. We thereby avoided a great deal of false positives that traditional searching would produce. This was demonstrated by validating the search and finding a practical precision of 98%. Generally, searches in large bibliographic databases have low precision [49]. One of the authors, a professional data analyst, conducted data cleaning to adjust for wrongly registered data. Furthermore, we collected data on publication dates from the CDSR, which is the primary source of Cochrane reviews. Therefore, we are confident that Cochrane reviews were correctly and sufficiently identified. However, our study also had some limitations. With large datasets, it is likely there were some miscategorized records. Based on our precision estimate and after data cleaning, we estimate this to be about 2% overall, but some areas may have had more than others. Despite the National Library of Medicine’s personal screening of records, we still found some protocols indexed as systematic reviews, indicating that the systematic review filter, while good, is not 100% perfect. Still, it is a more reliable method of retrieving systematic reviews than through searches and the most feasible method owing to the size of our sample. While searching PubMed ensured high external validity, there are undoubtedly more systematic reviews produced in the period indexed in other databases. However, correcting for this bias in our results would only exaggerate the conclusion that systematic reviews are increasing rapidly and constitute an increasingly larger portion of all systematic reviews compared with Cochrane reviews. On the other hand, a small portion of the increase in systematic reviews may be attributed to the post-indexation of older records and journals in PubMed. We do not believe this potential bias would impact the overall conclusions. Generally, we are confident that the assumed miscategorized data would not change the overall conclusions. This study cannot address the quality of the included systematic reviews or if there is an overlap of the research question of these systematic reviews, thus, whether unnecessary systematic reviews are being published.

It is unclear why Cochrane reviews were being published less frequently in the past 10 years. As Cochrane reviews are expected to be updated and despite them not always being updated frequently [50], we expected the number to increase given the combination of new Cochrane reviews being published and older ones being updated. It may be favorable to have more Cochrane reviews published, as they are generally of high quality owing to their thorough peer review process and rigorous methodology [27–32]. Some of the decrease in publication of Cochrane reviews may be due to them generally taking a long time to complete [21–23] and including many procedural and methodological demands [9, 10]. Future studies exploring why Cochrane reviews were being published less frequently are warranted. The continually increasing publication rates of systematic reviews may risk an information overload, where stakeholders cannot reasonably stay orientated and updated with new literature. It is, however, unclear to which degree the increase in publication of systematic reviews results from overlapping or redundant reviews being published, or if the new studies, in general, are of value to the research community. Further research into field overlap and the methodological quality of newer systematic reviews may clarify this.

In conclusion, the publication of systematic reviews has increased rapidly in the past 30 years, while fewer Cochrane reviews have been published since 2015. Especially Chinese first authors conducted many systematic reviews through the last 10 years. For the same period, gold open access journals were the largest publishers of systematic reviews. It may be favorable to have more Cochrane reviews published, as these are generally of higher quality than systematic reviews. Furthermore, further research regarding metrics related to research waste may clarify questions regarding the utility and value of systematic reviews throughout time.

### Supplementary Information

**Additional file 1.** Journals publishing non-Cochrane systematic reviews.

## Data Availability

Data were all retrieved from public databases and may be retrieved by researchers as described in the methods section of this manuscript. Alternatively, data are available from the authors upon reasonable request.

## Appendix

### RECORD reporting guideline checklist. The RECORD statement—a checklist of items, extended from the STROBE statement, that should be reported in observational studies using routinely collected health data

**Table:**

	**Item No**	**STROBE items**	**Location in manuscript where items are reported**	**RECORD items**	**Location in manuscript where items are reported**
**Title and abstract**					
	1	(a) Indicate the study’s design with a commonly used term in the title or the abstract, (b) provide in the abstract an informative and balanced summary of what was done and what was found	Page 1–3	RECORD 1.1: The type of data used should be specified in the title or abstract. When possible, the names of the databases used should be included RECORD 1.2: If applicable, the geographic region and timeframe within which the study took place should be reported in the title or abstract RECORD 1.3: If linkage between databases was conducted for the study, this should be clearly stated in the title or abstract	Page 1–3
**Introduction**					
Background rationale	2	Explain the scientific background and rationale for the investigation being reported	Page 3–4		
Objectives	3	State-specific objectives, including any prespecified hypotheses	Page 4		
**Methods**					
Study design	4	Present key elements of study design early in the paper	Page 4		
Setting	5	Describe the setting, locations, and relevant dates, including periods of recruitment, exposure, follow-up, and data collection	Page 4–5		
Participants	6	(a) *Cohort study*—give the eligibility criteria, and the sources and methods of selection of participants. Describe methods of follow-up *Case–control study*—give the eligibility criteria, and the sources and methods of case ascertainment and control selection. Give the rationale for the choice of cases and controls *Cross-sectional study*—Give the eligibility criteria, and the sources and methods of selection of participants (b) *Cohort study*—for matched studies, give matching criteria and number of exposed and unexposed *Case–control study*—for matched studies, give matching criteria and the number of controls per case	Page 4–5	RECORD 6.1: The methods of study population selection (such as codes or algorithms used to identify subjects) should be listed in detail. If this is not possible, an explanation should be provided RECORD 6.2: Any validation studies of the codes or algorithms used to select the population should be referenced. If validation was conducted for this study and not published elsewhere, detailed methods and results should be provided RECORD 6.3: If the study involved the linkage of databases, consider the use of a flow diagram or other graphical display to demonstrate the data linkage process, including the number of individuals with linked data at each stage	Page 4–6
Variables	7	Clearly define all outcomes, exposures, predictors, potential confounders, and effect modifiers. Give diagnostic criteria, if applicable	Page 6	RECORD 7.1: A complete list of codes and algorithms used to classify exposures, outcomes, confounders, and effect modifiers should be provided. If these cannot be reported, an explanation should be provided	Page 7
Data sources/measurement	8	For each variable of interest, give sources of data and details of methods of assessment (measurement) Describe comparability of assessment methods if there is more than one group	Page 6		
Bias	9	Describe any efforts to address potential sources of bias	Page 5–6		
Study size	10	Explain how the study size was arrived at	Page 6		
Quantitative variables	11	Explain how quantitative variables were handled in the analyses. If applicable, describe which groupings were chosen, and why	Page 6		
Statistical methods	12	(a) Describe all statistical methods, including those used to control for confounding (b) Describe any methods used to examine subgroups and interactions (c) Explain how missing data were addressed (d) *Cohort study*—if applicable, explain how loss to follow-up was addressed *Case–control study*—if applicable, explain how the matching of cases and controls was addressed *Cross-sectional study*—if applicable, describe analytical methods taking account of sampling strategy (e) Describe any sensitivity analyses	Page 6–7		
Data access and cleaning methods				RECORD 12.1: Authors should describe the extent to which the investigators had access to the database population used to create the study population RECORD 12.2: Authors should provide information on the data cleaning methods used in the study	Page 5–6
Linkage				RECORD 12.3: State whether the study included person-level, institutional-level, or other data linkage across two or more databases. The methods of linkage and methods of linkage quality evaluation should be provided	Page 5–6
**Results**					
Participants	13	(a) Report the numbers of individuals at each stage of the study (e.g., numbers potentially eligible, examined for eligibility, confirmed eligible, included in the study, completing follow-up, and analyzed) (b) Give reasons for non-participation at each stage (c) Consider the use of a flow diagram	Page 7	RECORD 13.1: Describe in detail the selection of the persons included in the study (i.e., study population selection) including filtering based on data quality, data availability, and linkage. The selection of included persons can be described in the text and/or by means of the study flow diagram	Page 7
Descriptive data	14	(a) Give characteristics of study participants (e.g., demographic, clinical, social) and information on exposures and potential confounders (b) Indicate the number of participants with missing data for each variable of interest (c) *Cohort study*—summarize follow-up time (e.g., average and total amount)	Page 7–12		
Outcome data	15	*Cohort study*—Report numbers of outcome events or summary measures over time *Case–control study*—report numbers in each exposure category, or summary measures of exposure *Cross-sectional study*—report numbers of outcome events or summary measures	Page 8–14		
Main results	16	(a) Give unadjusted estimates and, if applicable, confounder-adjusted estimates and their precision (e.g., 95% confidence interval). Make clear which confounders were adjusted for and why they were included (b) Report category boundaries when continuous variables were categorized (c) If relevant, consider translating estimates of relative risk into absolute risk for a meaningful time period	Page 8–14		
Other analyses	17	Report other analyses done—e.g., analyses of subgroups and interactions, and sensitivity analyses	Page 11–13		
**Discussion**					
Key results	18	Summarize key results with reference to study objectives	Page 14		
Limitations	19	Discuss the limitations of the study, taking into account sources of potential bias or imprecision. Discuss both the direction and magnitude of any potential bias	Page 16–17	RECORD 19.1: Discuss the implications of using data that were not created or collected to answer the specific research question(s). Include discussion of misclassification bias, unmeasured confounding, missing data, and changing eligibility over time, as they pertain to the study being reported	Page 16–17
Interpretation	20	Give a cautious overall interpretation of results considering objectives, limitations, multiplicity of analyses, results from similar studies, and other relevant evidence	Page 17–18		
Generalisability	21	Discuss the generalisability (external validity) of the study results	Page 17–18		
**Other information**					
Funding	22	Give the source of funding and the role of the funders for the present study and, if applicable, for the original study on which the present article is based	Page 19		
Accessibility of protocol, raw data, and programming code				RECORD 22.1: Authors should provide information on how to access any supplemental information such as the study protocol, raw data, or programming code	Page 19

## Abbreviations

MOOSE: Meta-analyses of Observational Studies in Epidemiology
PRISMA: Preferred Reporting Items for Systematic reviews and Meta-Analyses
CDSR: Cochrane Database of Systematic Reviews
DOI: Digital Object Identifier
NLM: National Library of Medicine
ISSN: International Standard Serial Number
