# Supplementary material for: Comparative analysis of Cochrane and non-Cochrane reviews over three decades
Source: Syst Rev. 2024 May 2;13:120. doi: 10.1186/s13643-024-02531-2 (PMC11064235; doi:10.1186/s13643-024-02531-2)
Supplement: Supplementary file 1 — Additional file 1. Journals publishing non-Cochrane systematic reviews. [file 13643_2024_2531_MOESM1_ESM.docx]

## Additional File 1

**Journals publishing non-Cochrane systematic reviews.**

**National Library of Medicine (NLM) abbreviations and International Standard Serial Number (ISSN) of journals with most published non-Cochrane systematic reviews based on ISSN expressed as: total number (%).**

| Rank | 1993–2002  (n=2,522) | | 2003–2012  (n=29,135) | | 2013–2022  (n=199,945) | | Total  (n=231,602) | |
| --- | --- | --- | --- | --- | --- | --- | --- | --- |
| #1 | BMJ (print)  0959-8138 | 95 (3.8) | JBI Libr Syst Rev  1838-2142 | 535 (1.8) | PloS One  1932-6203 | 4,404 (2.2) | PLoS One  1932-6203 | 4,680 (2.0) |
| #2 | Health Technol Assess  1366-5278 | 91 (3.6) | BMJ Clin Evid  1752-8526 | 452 (1.6) | Medicine (Baltimore)  1536-5964 | 2,263 (1.1) | Int J Environ Res Public Health  1660-4601 | 2,278 (1.0) |
| #3 | JAMA  0098-7484 | 31 (1.2) | BMJ (electronic)  1756-1833 | 298 (1.0) | Int J Environ Res Public Health  1660-4601 | 2,262 (1.1) | Medicine (Baltimore)  1536-5964 | 2,270 (1.0) |
| #4 | Br J Gen Pract  0960-1643 | 31 (1.2) | PLoS One  1932-6203 | 276 (0.9) | BMJ Open  2044-6055 | 1,735 (0.9) | BMJ Open  2044-6055 | 1,765 (0.8) |
| #5 | Lancet  0140-6736 | 30 (1.2) | Ann Intern Med  1539-3704 | 170 (0.6) | Nutrients  2072-6643 | 1,234 (0.6) | Nutrients  2072-6643 | 1,237 (0.5) |
| #6 | BMJ (electronic)  1756-1833 | 27 (1.1) | Spine  1528-1159 | 157 (0.5) | J Clin Med  2077-0383 | 1,040 (0.5) | J Clin Med  2077-0383 | 1,040 (0.4) |
| #7 | Spine  0362-2436 | 25 (1.0) | Health Technol Assess  2046-4924 | 154 (0.5) | Front Oncol  2234-943X | 978 (0.5) | Front Oncol  2234-943X | 978 (0.4) |
| #8 | Thorax  0040-6376 | 22 (0.9) | Obes Rev  1467-789X | 136 (0.5) | Sci Rep  2045-2322 | 927 (0.5) | Sci Rep  2045-2322 | 927 (0.4) |
| #9 | Br J Surg  0007-1323 | 21 (0.8) | BMC Public Health  1471-2458 | 134 (0.5) | World Neurosurg  1878-8769 | 799 (0.4) | BMC Public Health  1471-2458 | 829 (0.4) |
| #10 | Arch Intern Med  0003-9926 | 21 (0.8) | Aliment Pharmacol Ther  1365-2036 | 123 (0.4) | Cureus  2168-8184 | 775 (0.4) | World Neurosurg  1878-8769 | 802 (0.3) |
